# Supplementary material for: Molecular Evolution of CatSper in Mammals and Function of Sperm Hyperactivation in Gray Short-Tailed Opossum
Source: Cells. 2021 Apr 29;10(5):1047. doi: 10.3390/cells10051047 (PMC8147001; doi:10.3390/cells10051047)
Supplement: Supplementary file 1 [file cells-10-01047-s001.zip › Hwang_et_al_2021_Cells_Supplementary_Figures.pdf]

Supplementary Materials

Molecular Evolution of CatSper in Mammals and Function of Sperm Hyperactivation in Gray Short-Tailed Opossum

Jae Yeon Hwang[1,*], Jamie Maziarz[2,3], Günter P. Wagner[2,3,4] and Jean-Ju Chung[1,4*]

1 Department of Cellular and Molecular Physiology, Yale School of Medicine, New Haven, CT, 06510, USA
2 Department of Ecology and Evolutionary Biology, Yale University, New Haven, CT, 06520, USA
3 Yale Systems Biology Institute, Yale University, West Haven, CT, 06516, USA
4 Department of Obstetrics, Gynecology and Reproductive Sciences, Yale School of Medicine, New Haven,CT, 06510, USA
* Correspondence: jaeyeon.hwang@yale.edu (J.Y.H.) and jean-ju.chung@yale.edu (J.-J.C.)

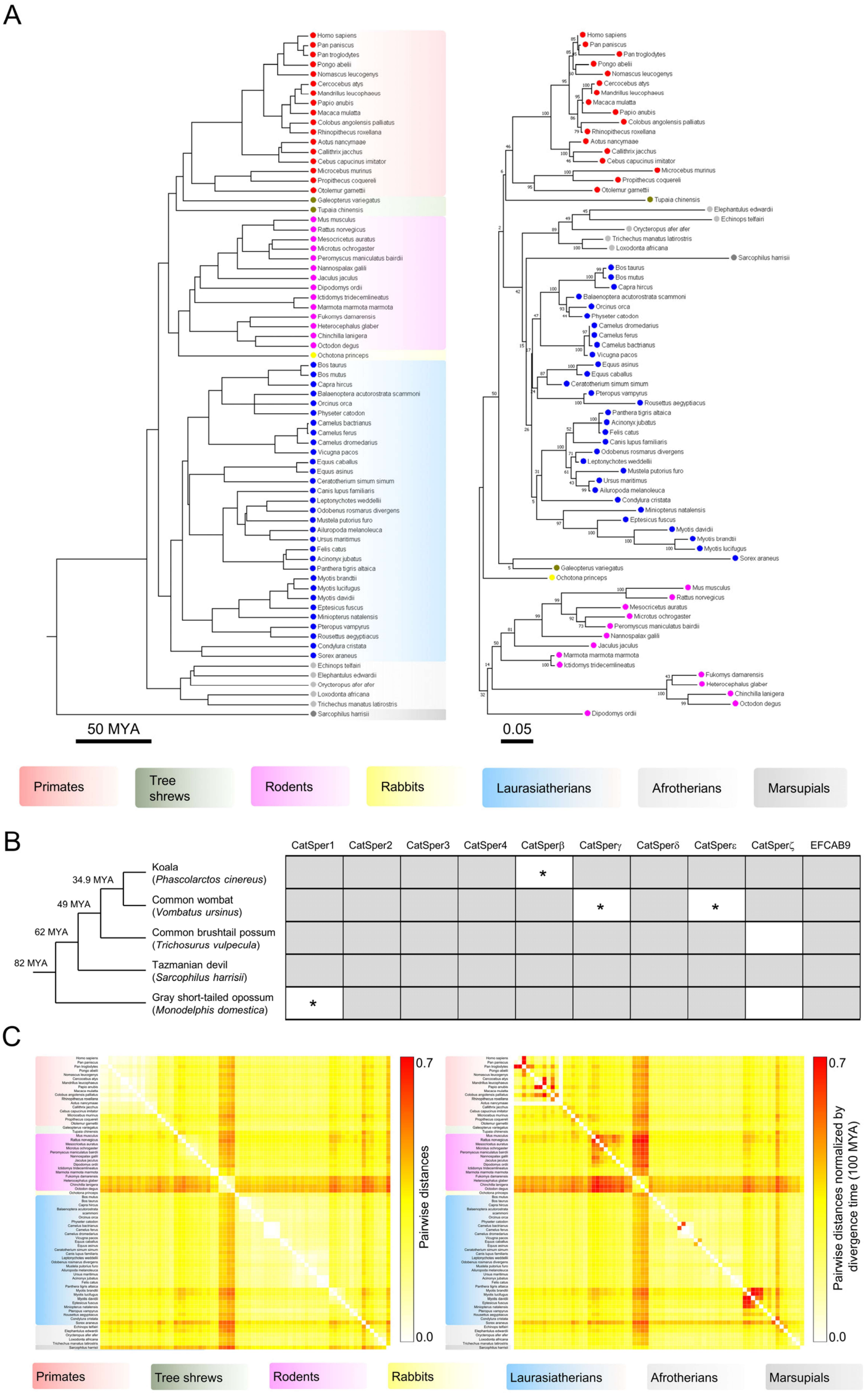
A
50 MYA
0.05
Primates
Tree shrews
Rodents
Rabbits
Laurasiatherians
Afrotherians
Marsupials
B
CatSper1
CatSper2
CatSper3
CatSper4
CatSperβ
CatSperγ
CatSperδ
CatSperε
CatSperζ
EFCAB9
34.9 MYA
49 MYA
62 MYA
82 MYA
Koala (Phascolarctos cinereus)
Common wombat (Vombatus ursinus)
Common brushtail possum (Trichosurus vulpecula)
Tazmanian devil (Sarcophilus harrisii)
Gray short-tailed opossum (Monodelphis domestica)
*
C
0.7
0.0
Pairwise distances
Pairwise distances normalized by divergence time (100 MYA)
Primates
Tree shrews
Rodents
Rabbits
Laurasiatherians
Afrotherians
Marsupials

Figure 1. Comparative protein sequ 29 ence analyses of CatSper components in placental mammals. (**A**) Phylogenetic trees of mammals were constructed using divergence time of each species based on fossil records (*left*, rooted, scale = 50 Million Years Ago, MYA) and maximum likelihood analysis of the concatenated protein sequences of ten CatSper subunits (CatSper1-2-3-4-β-γ-δ-ε-ζ-EFCAB9) (*right*, unrooted, scale = 0.05 pairwise distance). (B) Annotation of Marsupial CatSper subunits. Annotated marsupial CatSper subunits in NCBI Gene database are represented as heatmaps. All ten CatSper components are annotated for the Tasmanian devil. Presence or absence of the annotated components were marked with filled or empty cells. Empty cells with asterisks indicate the annotation of CatSper subunit37 like proteins in gray short-tailed opossum (CatSper1), koala (CatSperβ), and common wombat (CatSperγ and CatSperε). Divergence time of each species are marked (*left*). The phylogenic tree is unscaled. (**C**) Pairwise distance analysis of CatSper orthologs of between-species in mammals. Raw pairwise distances (*left*) and the values normalized by divergence time between two species (*right*) are represented by heatmaps. 100 MYA was set to 1 for the normalization. The values over 0.7 were set to 0.7 (red).

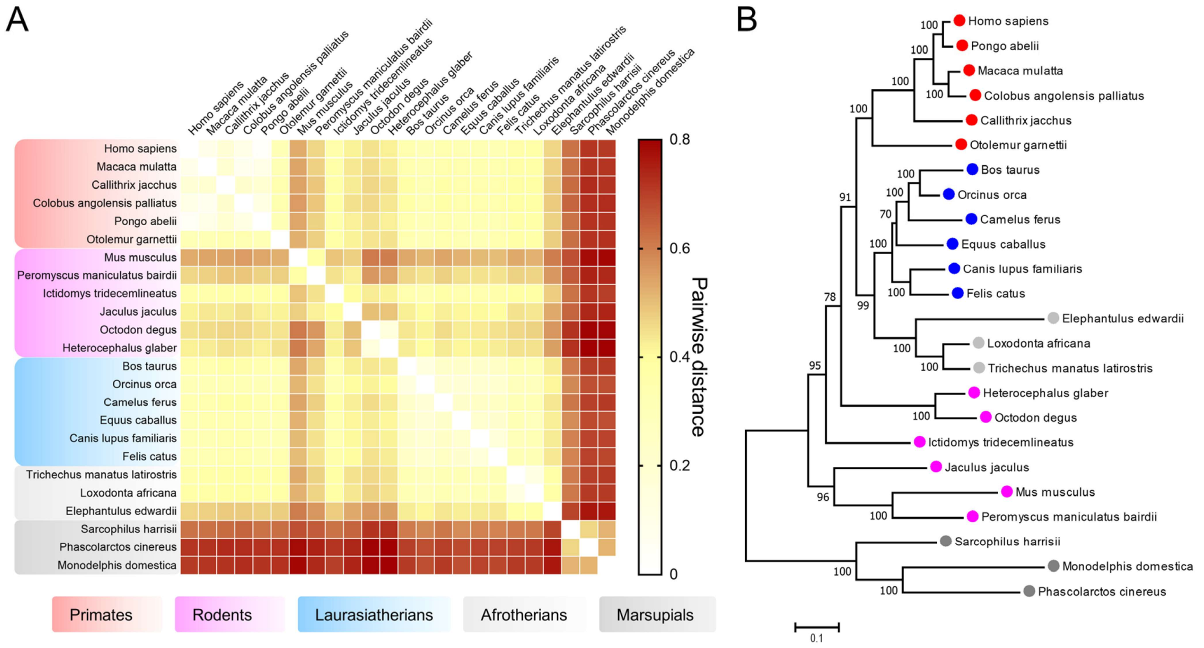

Figure 2. Marsupial CatSper subunits sequences are homologues. Protein sequences of CatSper subunits, except CatSperζ, were concatenated (CatSper1-2-3-4-β-γ-δ-ε-ζ-EFCAB9) and aligned to analyze pairwise distances (A) and phylogeny (B). The concatenated CatSper sequences from three marsupials, Tasmanian devil (*Sarcophilus harrisii*), koala (*Phascolarctos cinereus*), and gray short-tailed opossum (*Monodelphis domestica*), are divergent to those from eutherians (A) and clustered together in phylogenic analyses (B). The clades of each species are marked with colored-circles in the phylogenic tree; primates, red; rodents, magenta; Laurasiatheians, blue; Afrotherians, light gray; Marsupials, dark gray. Scale bar = genetic distance (B).

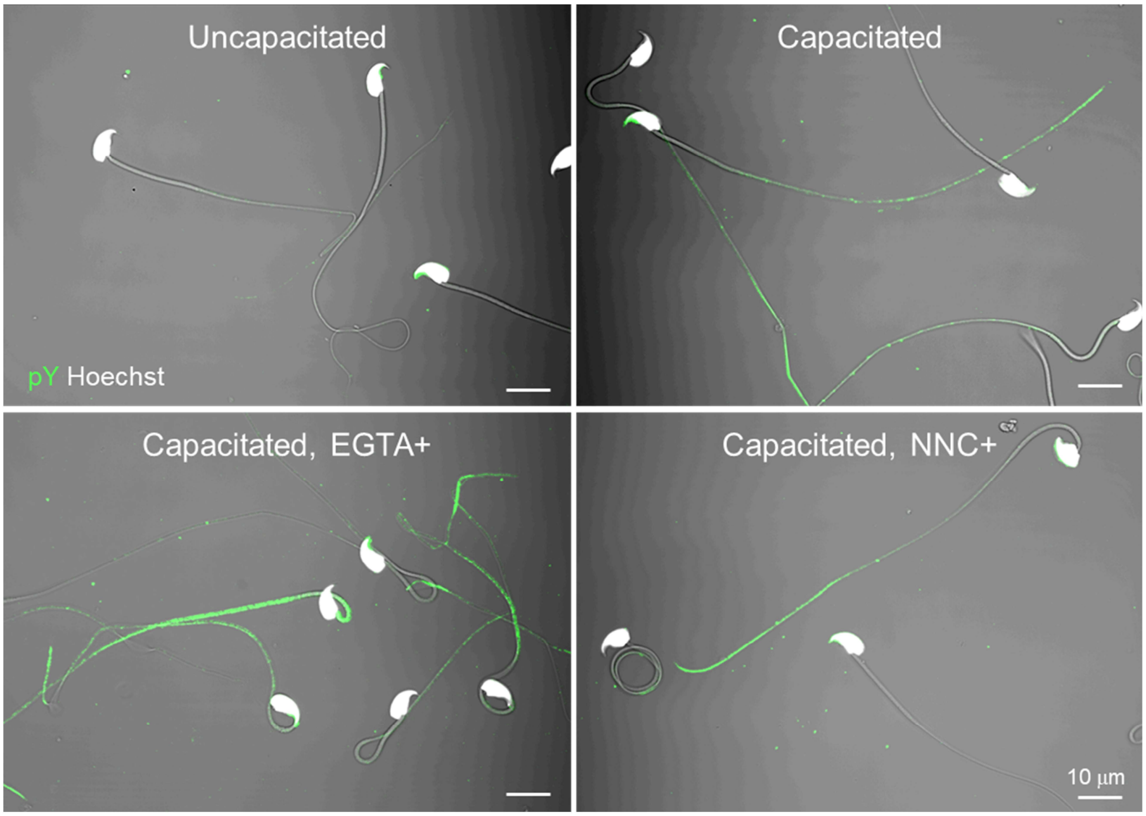

Figure 3. Impaired Ca^{2+} entry enhances global tyrosine phosphorylation (pY) during capacitation in mouse sperm cells. Confocal images of immunostained pY in mouse sperm cells are shown. Mouse sperm cells were capacitated in HTF medium supplemented with 2.5 mM EGTA (EGTA+) or 10 μM NNC 55-0396 (NNC+). Merged fluorescence and corresponding DIC images are shown. Sperm heads were stained with Hoechst.

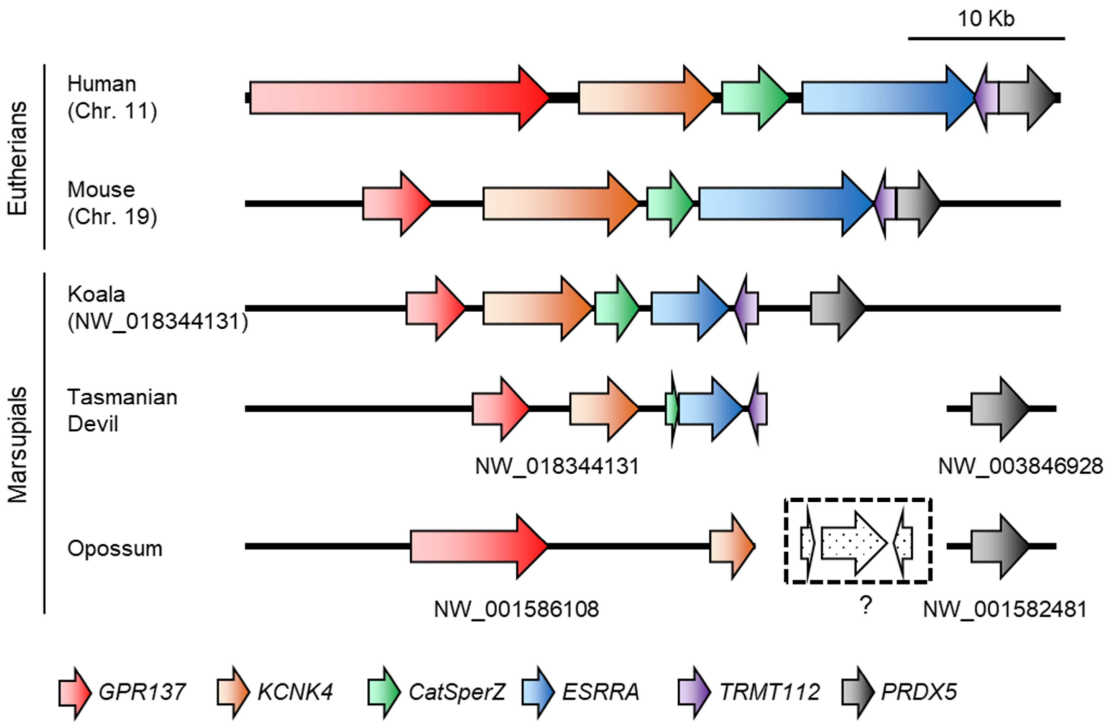

Figure 4. CatSperζ orthologs are conserved in marsupials. Diagrams indicate structures of the genomic region encoding CatSper and neighboring genes from eutherians and marsupials. *CatSperz* (green), *ESRRA* (blue), and *TRMT112* (purple) are annotated in two of the marsupials examined, Koala and Tasmanian devil, but not in the grey short-tailed opossum. Comparison of the genomic region shows conserved synteny, suggesting the ortholog of CatSperζ is also likely to be conserved in opossum. Accession numbers below the genomic structure indicate assembled scaffolds of Tasmanian devil (NW_018344131 and NW_003846928) and gray short-tailed opossum (NW_001586108 and NW_001582481).

Table S1. Species and CatSper orthologs information for sequence analyses.

Table S2. Primer pairs for qRT-PCR used in this study.

Video S1. Flagella movement of the head-tethered opossum sperm. Tail movement of the opossum sperm before (*left*) and after (*middle* and *right*) inducing capacitation were recorded at 37 °C in H-HTF medium supplemented with 0.5% methylcelluose. Each video is played at 100 fps (1/2 speed).

Video S2. Movements of free-swimming opossum sperm. Movements of paired (*left*) and single sperm (*right*) were recorded before (*top*) and after (*bottom*) inducing capacitation in a 37 °C H-HTF medium containing 0.5% methylcellulose. Each video is played at 100 fps. **Video S3**. Flagella movement of the opossum sperm capacitated under Ca^{2+}-chelated condition with EGTA. Tail movement of the head-tethered sperm cells were recorded in H-HTF medium with 0.5% methylcellulose at 37 °C. Each video is played at 100 fps (1/2 speed).

Video S4. Free-swimming of the opossum sperm capacitated with EGTA. Movements of the swimming sperm capacitated under Ca^{2+}-chelated condition were recorded in a H-HTF medium with 0.5% methylcellulose at 37 °C. Each video is played at 100 fps.

Video S5. Flagella movement of the opossum sperm treated with CatSper-inhibitor, NNC 55-0396 during capacitation. Sperm capacitated with NNC 55-0396 were head tethered and their tail movements were recorded at 37 °C in a H-HTF medium supplemented with 0.5% methylcellulose. Videos are played at 100 fps (1/2 speed).

Video S6. Free-swimming of the opossum sperm capacitated with NNC 55-0369 (NNC). Movements of the free-swimming opossum sperm capacitated in HTF medium supplemented with NNC were recorded at 37 C in a 0.5% methylcellulose-containing H-HTF medium. Videos are played at 100 fps.
